# How many chronic myeloid leukemia patients who started a frontline second‐generation tyrosine kinase inhibitor have to switch to a second‐line treatment? A retrospective analysis from the monitoring registries of the italian medicines agency (AIFA)

**DOI:** 10.1002/cam4.3071

**Published:** 2020-04-22

**Authors:** Massimo Breccia, Pier Paolo Olimpieri, Odoardo Olimpieri, Fabrizio Pane, Alessandra Iurlo, Paolo Foggi, Alessia Cirilli, Antonietta Colatrella, Marcello Cuomo, Lucia Gozzo, Valentina Summa, Paolo Corradini, Pierluigi Russo, the AIFA’s Monitoring Registries Group

**Affiliations:** ^1^ Department of Translational and Precision Medicine Az. Policlinico Umberto I‐Sapienza University Rome Italy; ^2^ Italian Medicines Agency Rome Italy; ^3^ Università degli Studi di Napoli Federico II Naples Italy; ^4^ Foundation IRCCS Ca' Granda Ospedale Maggiore Policlinico Milano Italy; ^5^ Università degli Studi di Milano & Divisione Ematologia Fondazione IRCCS Istituto Nazionale dei Tumori di Milano Milano Italy

**Keywords:** chronic myeloid leukemia, failure, intolerance, second‐generation TKIs

## Abstract

The frequency of patients who switch to a second‐line therapy from a frontline second‐generation (2gen) tyrosine kinase inhibitor (TKI) such as dasatinib and nilotinib, is still substantially unknown. We retrospectively investigated a large series of chronic phase chronic myeloid leukemia (CP‐CML) patients initially treated with 2gen TKIs monitored through the Italian Medicines Agency (AIFA Agenzia Italiana del farmaco) registries. Overall, 2420 patients were analyzed over a period of 6 years. One hundred and fifty‐seven patients (16.3%) treated with dasatinib and 164 treated with nilotinib (11.3%) have switched to another drug, with an overall frequency of 13.2%. In the dasatinib cohort, 39.4% of patients changed treatment for failure and 36.3% for intolerance as compared to 45.7% and 27.4% respectively in the nilotinib cohort. Overall, the median time to switch due to resistance was 293 days, whereas it was 317 days in case of intolerance. Resistance was observed mainly in younger male patients with high‐risk features, while intolerance was not related to any baseline parameter. After resistance/intolerance to nilotinib, the majority of patients switched to dasatinib (53.8%) whereas in case of frontline dasatinib to ponatinib (43.2%). To the best of our knowledge these data provide the first report on the frequency of discontinuation of frontline 2gen TKIs and on the main causes and pattern of choice to a second‐line therapy in the real‐life setting.

## Graphical abstract

Overall, 2420 patients were analyzed over a period of 6 years. One hundred and fifty‐seven patients (16.3%) treated with dasatinib and 164 treated with nilotinib (11.3%) have switched to another drug, with an overall frequency of 13.2%. In the dasatinib cohort, 39.4% of patients changed treatment for failure and 36.3% for intolerance as compared to 45.7% and 27.4% respectively in the nilotinib cohort. Resistance was observed mainly in younger male patients with high‐risk features, while intolerance was not related to any baseline parameter. To the best of our knowledge these data provide the first report on the frequency of discontinuation of frontline 2gen TKIs and on the main causes and pattern of choice to a second‐line therapy in the real‐life setting.

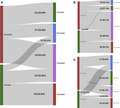

## 1 INTRODUCTION

The treatment of chronic myeloid leukemia (CML) has drastically changed since the introduction of imatinib, the first‐generation tyrosine kinase inhibitor (TKI). Imatinib induced more than 85% of complete cytogenetic response (CCyR) with a major molecular response (BCR‐ABL1 ratio < 0.1% according to International Scale or 3 log‐reduction) in approximately 60% of patients.^1^, ^2^ However, despite these excellent results, more than 30% of treated subjects experienced treatment failure either for resistance or intolerance and had to be switched to a second‐line therapy.^3^ Frontline treatment with second‐generation TKIs (2gen TKIs), namely dasatinib and nilotinib, has further improved the outcome: although no differences in terms of overall survival have been detected as compared to imatinib, both these drugs induced faster and deeper molecular responses, decreasing the number of patients experiencing a progression to advanced phases of the disease.^4^, ^5^ Despite the confirmed long‐term results, some patients still experienced either failure or severe intolerance to 2gen TKIs requiring therefore a subsequent therapy. According to the only published experience on the outcome of patients resistant/intolerant to a frontline 2gen TKI reported by MD Anderson Cancer Center (MDACC) out of 218 patients treated with dasatinib or nilotinib after a median follow‐up of 23 months, 40 patients (18%) discontinued therapy, 25 initially treated with nilotinib (21% of all treated with nilotinib) and 15 (15%) initially treated with dasatinib. The majority of patients switched to a subsequent line for intolerance (16 subjects) and only a minor percentage for resistance (5 patients) or for progression (4 patients). Eleven subjects received imatinib as second‐line therapy and only two patients switched to third‐generation ponatinib.^6^ Considering the still persisting paucity of data on frequency and causes of discontinuation from frontline 2gen TKIs and on second‐line therapy selection, the aim of this study is to provide a real‐life picture on these crucial issues based on a large series of Italian patients from AIFA registries.

## 2 PATIENTS AND METHODS

For this analysis, we collected data from AIFA registries of all newly diagnosed chronic phase CML adult Italian patients treated with frontline dasatinib or nilotinib from January 2013 to December 2018. The web‐system allowed the storing and monitoring of clinical characteristics of patients eligible for treatment with 2gen TKIs according to prespecified criteria. Registered parameters for all TKIs were therapeutic drug indication, baseline characteristics (including age and Sokal score), patient outcome, treatment duration, principal reason for treatment discontinuation, occurrence of adverse reactions. According to Italian laws monitoring of these parameters does not require any informed consent or formal approval from ethical committees. Any included patient did receive, however, information about the purposes of the monitoring. Dasatinib and nilotinib eligibility form included demographic data (place of birth, age, and sex) and the Sokal risk class.^7^ Prescription forms recorded the date of prescription, dose administered, and whether adverse reactions had occurred since the last prescription. Finally, the end of treatment form collected the reasons for treatment discontinuation. Time to treatment discontinuation (TTD) was defined as the time occurring between the initial prescription and the date of treatment discontinuation for any cause, including death and lost to follow‐up. A patient was defined “lost to follow‐up” in the absence of any prescription or re‐evaluation for at least 180 days after the last registry entry. Switches to a different TKI were assessed considering also bosutinib and ponatinib as destination treatment, by checking the relative AIFA monitoring registries on the basis of a patient unique identifier. Since imatinib was not actively monitored by AIFA, some uncertainties on subsequent switch to first‐generation TKI remains for the patients that have been lost to follow‐up. The time interval between the end of the first TKI treatment and the beginning of the second line was also evaluated, in order to rule out the possibility that other unmonitored therapy might have been administered between two subsequent recorded treatments.

## 3 RESULTS

From January 2013 to December 2018, 2.420 patients with newly diagnosed CP‐CML treated with frontline 2gen TKIs were recorded into the AIFA registries. Of them, 964 patients (39.8%) were treated with dasatinib and 1456 (60.2%) with nilotinib. A change of therapy was recorded in 321 subjects (13.2%), 157 (16.3%) of the dasatinib cohort, and 164 (11.3%) of the nilotinib cohort. The median time for switch was 354 days, with a median of 386 days for dasatinib and 323 days for nilotinib‐treated patients. Overall, 6.9% of patients switched within 365 days from start of treatment, 7.6% of the dasatinib cohort and 6.5% of the nilotinib cohort. Baseline features of CML patients treated with 2gen TKIs who change therapy are reported in Table 1. Overall, at the time of first prescription the median age was 54 years (range 21‐83) with a prevalence of male patients (59.8%). The majority of patients were aged less than 65 years (76%) with only 77 older subjects (> or equal to 65 years, 23.9%). According to Sokal risk, 103 (32%) patients were classified as low risk, 118 (36.7%) as intermediate and 100 (31.1%) as high risk (Table 1). A comparison between the dasatinib and nilotinib populations revealed a significant difference in terms of Sokal risk (*P* < .00001) and age at presentation, with patients treated with nilotinib being significantly younger than patients who received dasatinib (*P* < .00001). As far as the main reasons for discontinuation are concerned, 137 subjects (42.68%) were considered as treatment failure, 102 (31.78%) as intolerant, whereas 51 patients (15.89%) switched for nondrug‐related reasons and in 31 patients (9.6%) the cause is missing.

**Table 1:** Baseline features of whole cohort

	Dasatinib	Nilotinib	Overall
	(N = 157)	(N = 164)	(N = 321)
Gender—no. (%)			
Female	63 (40.13%)	66 (40.24%)	129 (40.19%)
Male	94 (59.87%)	98 (59.76%)	192 (59.81%)
Age			
Median—y	57	51	54
Range—y	21‐82	21‐83	21‐83
<65 y	108 (68.79%)	136 (82.93%)	244 (76.01%)
≥65 y	49 (31.21%)	28 (17.07%)	77 (23.99%)
Sokal score			
Low‐risk	41 (26.11%)	62 (37.80%)	103 (32.09%)
Intermediate‐risk	63 (40.13%)	55 (33.54%)	118 (36.76%)
High‐risk	53 (33.76%)	47 (28.66%)	100 (31.15%)
Switch	157 (16.3%)	164 (11.3%)	321 (13.27%)
Switch per 1000 y of treatment	69.6	45.7	55.0
Mean (median) days at switch	467 (386)	393 (323)	430 (354)
Switch within 365 d from treatment start	73 (7.6%)	94 (6.5%)	167 (6.9%)

Considering the 137 patients who changed therapy for resistance/progression (Table 2), the overall median time to the switch was 293 days, 286 days for dasatinib and 293 days for nilotinib‐treated patients. Fifty‐nine percent of patients switched within 365 days from treatment start. This cohort was represented mainly by male patients (63.5%), median age 52 years, being only 25 of them (18.2%) aged ≥ 65 years. The majority of patients were classified as having at presentation a high Sokal risk (40.8%) or intermediate risk (33.5%) with only 25.5% being classified as low risk (Table 2).

**Table 2:** Resistant cohort: baseline features

	Dasatinib	Nilotinib	Overall
	(N = 62)	(N = 75)	(N = 137)
Gender—no. (%)			
Female	21 (33.87%)	29 (38.67%)	50 (36.50%)
Male	41 (66.13%)	46 (61.33%)	87 (63.50%)
Age			
Median—y	57	51	52
Range—y	24‐82	21‐83	21‐83
<65 y	46 (74.19%)	66 (88.00%)	112 (81.75%)
≥65 y	16 (25.81%)	9 (12.00%)	25 (18.25%)
Sokal score			
Low‐risk	12 (19.35%)	23 (30.67%)	35 (25.55%)
Intermediate	23 (37.10%)	23 (30.67%)	46 (33.58%)
High‐risk	27 (43.55%)	29 (38.67%)	56 (40.88%)
Mean (Median) days at switch	386 (286.5)	335 (293)	358 (293)
Switch within 365 d from treatment start	34 (54.8%)	47 (62.7%)	81 (59.1%)

One hundred and two patients switched for intolerance after a median time of 317 days, 386 for dasatinib and 189 for nilotinib‐treated subjects (Table 3). We can speculate about the short time observed in nilotinib‐treated patients implying the possible related metabolic side effects which have required discontinuation. In this cohort there was a slight difference in terms of gender with 51.9% male and 48.1% of female subjects. The median age was 57 years (range 22‐80) with 35% of patients aged ≥ 65 years; no difference among Sokal risk classification at baseline was detected, being 29.4% low risk, 45% intermediate and 25.4% as high risk (Table 3).

**Table 3:** Intolerant cohort: baseline features

	Dasatinib	Nilotinib	Overall
	(N = 57)	(N = 45)	(N = 102)
Gender—no. (%)			
Female	25 (43.86%)	24 (53.33%)	49 (48.04%)
Male	32 (56.14%)	21 (46.67%)	53 (51.96%)
Age			
Median—r	60	51	57
Range—y	32‐80	22‐78	22‐80
<65 y	32 (56.14%)	34 (75.56%)	66 (64.71%)
≥65 y	25 (43.86%)	11 (24.44%)	36 (35.29%)
Sokal score			
Low‐risk	15 (26.32%)	15 (33.33%)	30 (29.41%)
Intermediate‐risk	28 (49.12%)	18 (40.00%)	46 (45.10%)
High‐risk	14 (24.56%)	12 (26.67%)	26 (25.49%)
MEAN (median) days at switch	517 (386)	327 (189)	434 (317)
Switch within 365 d from treatment start	26 (45.6%)	32 (71.1%)	58 (56.9%)

**Figure 1:**
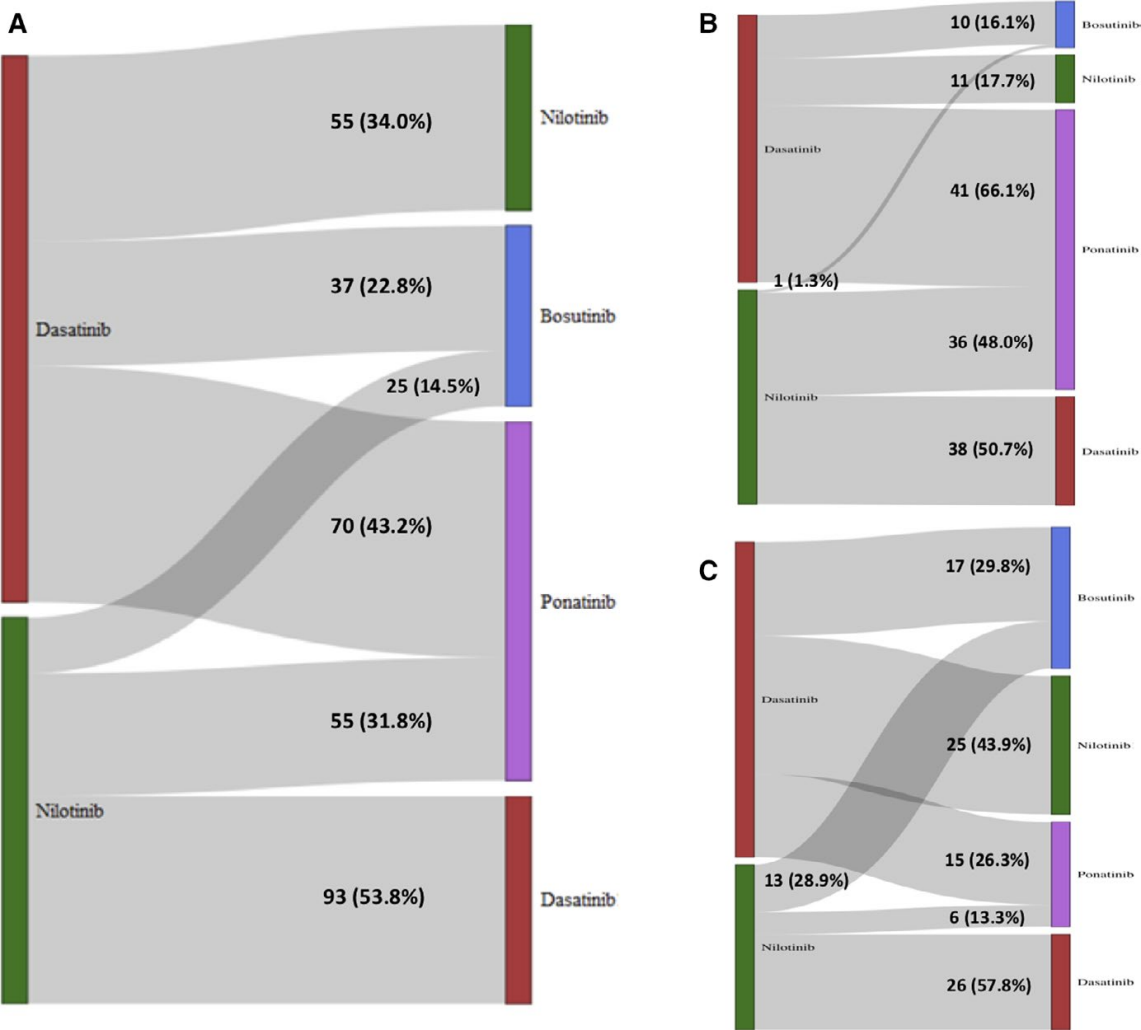
Second choice after discontinuation of first‐line dasatinib (in red) or nilotinib (in green) for any reasons (panel A), for treatment failure (panel B) or intolerance (panel C). Second TKI bosutinib and ponatinib are blue and violet colored, respectively

**Figure 2:**
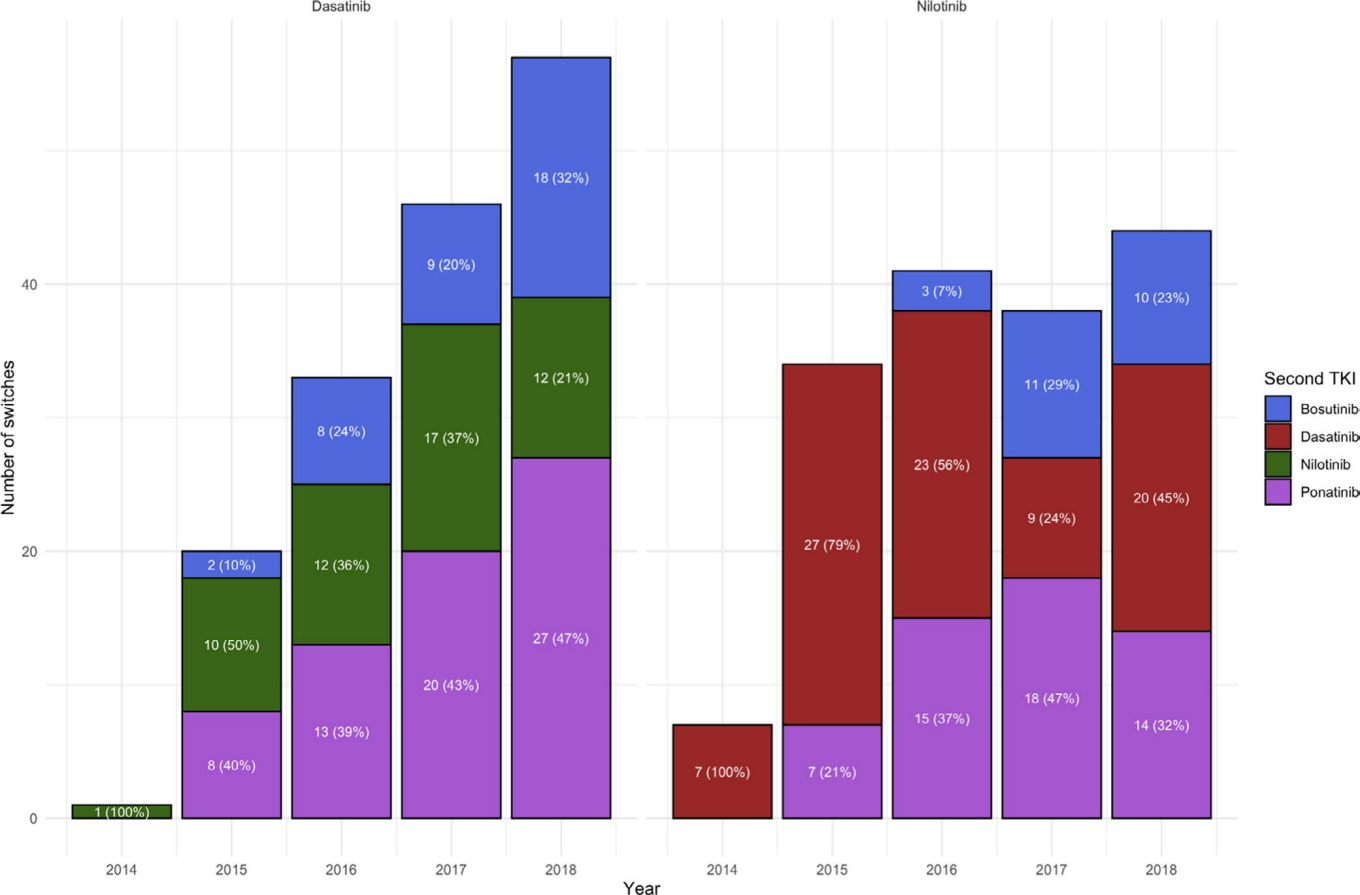
Choice of second drug reported year by year in a period of 5 y

Details on second‐line therapies after discontinuation of frontline dasatinib or nilotinib are reported in Figure 1. The majority of patients who changed from nilotinib, received as second‐line dasatinib (53.8%), ponatinib (31.8%), or in lower percentage bosutinib (14.5%) while from dasatinib the majority of subjects switched to ponatinib (43.2%), 34% to nilotinib and 22.8% to bosutinib. The choice of second line over time is represented in Figure 2.

## 4 DISCUSSION

Second‐generation TKIs used as frontline treatment increased the rate of faster and deeper molecular responses over time, reducing the possibility to experience a progression to advanced phase.^8^, ^9^ As with imatinib, some patients can experience resistance and/or intolerance to these drugs, but remains still largely unknown how many patients treated outside clinical trials discontinue the treatment permanently. Until now only a few publications reported the outcome of frontline 2gen TKI CML‐treated patients, in particular the SIMPLICITY trial^10^, ^11^ and the MDACC experience.^6^ The SIMPLICITY trial is an observational study performed in US and six European countries with the primary aim of investigating the frontline TKI use and management patterns in routine clinical practice. Twelve‐month follow‐up data are reported from October 2010 to September 2015; out of 1242 patients enrolled, 418 received dasatinib and 408 were treated with nilotinib.^10^ Treatment selection was based on the baseline comorbidity profile with a predominance for 2gen TKIs from 2014 onward. A comparison of Italian rate of discontinuation with the other European countries showed a rate of permanent discontinuation in Italy in the first 12 months of 8.9% for dasatinib cohort and 15.1% in the nilotinib‐treated cohort, whereas in the rest of European countries it was of 19.6% for dasatinib and 13.8% for nilotinib.^11^

While the median time of discontinuation in Italy was 4.6 and 5.5 months for dasatinib and nilotinib, respectively, in the remaining countries it was shorter being 4.4 months for dasatinib and 1.4 months for nilotinib. Intolerance was reported as the most frequent cause of first‐line TKI discontinuation while primary resistance was the second most common one. No statistically significant predictive factors have been identified in the Italian population, whereas in the rest of European countries female and patients treated with imatinib were more likely to discontinue the treatment. While the switch to a second line occurred mostly between 3 and 9 months for dasatinib‐treated patients similarly to what occurred for nilotinib, with imatinib the majority of patients switched earlier after 3 months of treatment.

As compared to this sponsored trial not representative of the actual real‐life setting due to the criteria for selection of patients, according to our data from AIFA registries in Italy 157 patients (16.3%) treated with frontline dasatinib and 164 treated with nilotinib (11.3%) have switched to another drug, with an overall incidence of 13.2%. Differently from what usually reported in clinical trials, the main reason to discontinue a frontline 2gen TKI seems to be resistance being 39.4% in the dasatinib‐treated cohort compared to 45.7% in nilotinib‐treated patients. Interestingly, the majority of patients were aged less than 65 years (76%) with only 77 (23.9%) older subjects; it can be speculated that 2gen TKIs were used prevalently in younger patients, probably due to specific safety profile and that probably the rate of discontinuation is significantly lower, if compared to sponsored trials, due to the absence of dose modification and discontinuation forced criteria. Overall, the Sokal risk stratification showed that the majority of patients who experienced resistance and failure were classified as having at presentation a high Sokal risk (40.8%) or intermediate risk (33.5%) with only 25.5% being classified as having a low Sokal risk. This is in line with the results of sponsored clinical trials showing an increased rate of events in high‐risk patients as compared to low Sokal risk, both in terms of resistance and rate of progression; no difference was observed in Sokal risk stratification in the intolerant cohort analyzed. Median time to discontinuation was 9.5 months for dasatinib and 9.7 months for nilotinib: these data, indeed, indicate a conservative approach of physicians when starting a frontline 2gen TKIs as compared to imatinib.^12^, ^13^ In fact, with this latter drug a possible switch occurs more frequently after only 3 months. A real‐life experience of outcome after discontinuation of 2gen TKIs was reported by MDACC group ^6^: out of 218 patients frontline treated with dasatinib and nilotinib 40 subjects (18%) discontinued the therapy, contrary to our experience, mostly for toxicity and only five patients for resistance; as in our study, the median time of discontinuation was 8 months. Overall, as per second choice of TKIs it seems that ponatinib is preferred after resistance, in particular after dasatinib failure (66% of patients switched from dasatinib to ponatinib) probably due to high potency and multitarget activity of the drug. Even after intolerance, ponatinib was chosen in 26% of patients after dasatinib and 13% after nilotinib, probably because it is possible to prescribe low dose of the drug. Since imatinib was not actively monitored by AIFA, some uncertainties on subsequent switch to first‐generation TKI remains for the patients that have been lost to follow‐up. Anyway, given the low percentage of these patients (about 4% of the overall population), the number of putative switches to imatinib might be considered to be very low.

Despite some limitations due to the retrospective nature of this investigation and the lack of some clinical data not required by the AIFA registries as for instance on the outcome of patients experiencing a switch of therapy, our study representative of the Italian real‐life setting showed that treatment discontinuation with frontline 2gen TKIs was relatively uncommon.

Further analyses are planned in order to acquire extensive data on patients treated with subsequent lines of therapy and subjects who attempt a discontinuation.

## CONFLICT OF INTEREST

MB received honoraria by Novartis, Incyte, Pfizer, Celgene; all the other authors have no conflict of interest.

## AUTHORS CONTRIBUTION

MB interpreted the data and wrote the manuscript. PPO, OO, PF, AC, AC, MC, LG, and VS collecting the data, finalized the statistical analysis and revised the final version. AI and FP collecting the data and revised the final version, PC and PR revised and approved the final version.
